# Curcumin restores mitochondrial functions and decreases lipid peroxidation in liver and kidneys of diabetic *db/db* mice

**DOI:** 10.1186/0717-6287-47-74

**Published:** 2014-12-22

**Authors:** María G Soto-Urquieta, Sergio López-Briones, Victoriano Pérez-Vázquez, Alfredo Saavedra-Molina, Gloria A González-Hernández, Joel Ramírez-Emiliano

**Affiliations:** Departamento de Ciencias Médicas, Universidad de Guanajuato, 20 de Enero 929, Col. Obregón, C.P. 37320, León, GTO, México; Instituto de Investigaciones Químico-Biológicas, Universidad Michoacana de San Nicolás de Hidalgo, Morelia, MICH, México; University of Texas Medical Branch at Galveston, Galveston, TX USA; Departamento de Biología, Universidad de Guanajuato, León, GTO, México

**Keywords:** Diabetes, Mitochondria, Curcumin, ATPase activity, Nitric oxide synthesis, Lipid oxidation

## Abstract

**Background:**

Nitrosative and oxidative stress play a key role in obesity and diabetes-related mitochondrial dysfunction. The objective was to investigate the effect of curcumin treatment on state 3 and 4 oxygen consumption, nitric oxide (NO) synthesis, ATPase activity and lipid oxidation in mitochondria isolated from liver and kidneys of diabetic *db/db* mice.

**Results:**

Hyperglycaemia increased oxygen consumption and decreased NO synthesis in liver mitochondria isolated from diabetic mice relative to the control mice. In kidney mitochondria, hyperglycaemia increased state 3 oxygen consumption and thiobarbituric acid-reactive substances (TBARS) levels in diabetic mice relative to control mice. Interestingly, treating *db/db* mice with curcumin improved or restored these parameters to normal levels; also curcumin increased liver mitochondrial ATPase activity in *db/db* mice relative to untreated *db/db* mice.

**Conclusions:**

These findings suggest that hyperglycaemia modifies oxygen consumption rate, NO synthesis and increases TBARS levels in mitochondria from the liver and kidneys of diabetic mice, whereas curcumin may have a protective role against these alterations.

## Background

Obesity and diabetes are serious and growing public health problems that result in reduced life expectancy and increased morbidity due to disease-specific vascular complications. Moreover, there is considerable evidence linking obesity and diabetes with oxidative stress and mitochondrial dysfunction in human and animal models. For instance, serum thiobarbituric acid-reactive substances (TBARS) were elevated, and serum total thiols and superoxide dismutase (SOD) activity were decreased in patients with metabolic syndrome compared with healthy subjects [1]. Likely, systemic oxidative stress was increased in obese children with and without metabolic syndrome [2], and reactive oxygen species (ROS) and malondialdehyde (MDA) were higher in diabetic subjects compared with control subjects [3]. With respect to adenosine-5′-triphosphate (ATP) synthesis, abdominal obesity was associated with reduced mitochondrial ATP and ROS production rates in skeletal muscle of men [4]. Moreover, diabetic patients had reduced ATP synthesis and elevated lipid contents in liver [5], as well as reduced ATP synthesis [6, 7]; and reduced expression of the α-subunit of ATP synthase [8] in skeletal muscle as compared to control subjects.

Mitochondria isolated from the livers of mice that were fed a high-fat diet (HFD) exhibit decreased state 3 respiration, decreased uncoupled respiration, and alterations in cytochrome *c* oxidase activity and mitochondrial membrane potential [9]. In the HFD-fed mice, there was accumulation of 3-nitrotyrosine and increased sensitivity to nitric oxide (NO)-dependent respiratory inhibition compared with non-obese controls.

To reduce oxidative stress caused by obesity and diabetes, dietary supplementation of antioxidants has been proposed. Different studies have shown that curcumin has antioxidant and anti-hyperglycaemic properties in diabetic and obese animal models [10–13]. It was reported that the activity of erythrocyte antioxidant enzymes superoxide dismutase and catalase were significantly higher (2- and 1.5-fold, respectively), and glutathione peroxidase activity was significantly lower (0.7 fold) in diabetic *db/db* mice than in nondiabetic db/+ mice. In the *db/db* mice, these enzyme activities were restored to basal values after curcumin treatment. Curcumin also significantly decreased MDA levels from 479.7 to 393.3 nmol/g haemoglobin in erythrocytes of these mice [12]. Furthermore, in PC12 cells that were exposed to 4-hydroxynonenal, curcumin treatment prevented changes in glutathione levels as well as mitochondrial oxidative damage and respiration and decreased the accumulation of carbonylated proteins and apoptosis [14].

We recently showed that curcumin treatment increased oxygen consumption and significantly decreased lipid and protein oxidation levels in liver mitochondria isolated from HFD-induced obese mice compared with those in the untreated obese mice [15]. In kidney mitochondria, curcumin treatment significantly increased oxygen consumption and decreased lipid and protein peroxidation levels in HFD-induced obese mice when compared with those in untreated obese mice. Curcumin treatment neither had any effect on body weight gain or on mitochondrial NO synthesis.

Therefore, we testing hypotheses of that dietary supplementation with curcumin improve indices of lipid oxidation, oxygen consumption rate, nitric oxide (NO) synthesis and ATPase activity in the liver and kidneys of diabetic *db/db* mice.

## Results

### Curcumin decreases body weight in db/db mice

**Figure 1:**
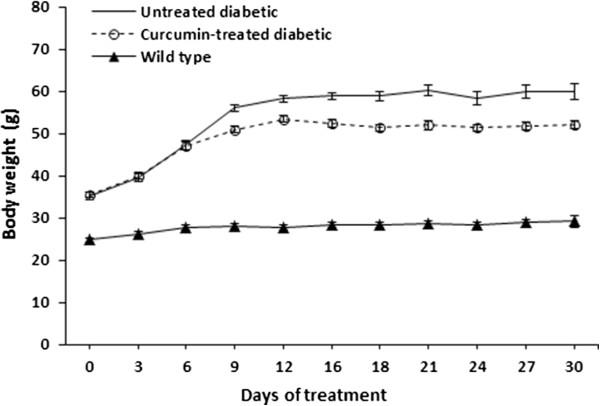
**Effect of hyperglycaemia and curcumin on the gain of body weight.** Mice were weighted every three days for one month. Wild type mice (n = 4); Untreated-diabetic mice (n = 4); Treated-diabetic mice (n = 9). Data are given as the means ± standard error of the mean (SEM).

To examine the effect of curcumin on the gain of body weight, the mice were weighted at the beginning of the curcumin treatment and no significant differences were observed between the untreated diabetic and curcumin-treated diabetic mice (35.3 ± 0.9 and 35.6 ± 0.5 g, respectively), both groups of diabetic mice were significantly heavier than wild type mice (Figure 1). At the end of the treatment, curcumin treatment significantly decreased the gain of body weight of the diabetic mice (52 ± 0.9 g) compared with diabetic mice that were not treated (60 ± 1.6 g). The diabetic mice, whether treated with curcumin or untreated, were significantly heavier than the wild type mice (29 ± 0.8 g).

### Curcumin decreases blood glucose levels in db/db mice

In untreated diabetic mice, blood glucose levels were higher than in the wild type mice (488.2 ± 73 vs. 152 ± 11 mg/ml, p < 0.01). Curcumin treatment decreased blood glucose levels in diabetic mice (196.3 ± 13 mg/ml) as compared to untreated diabetic mice (p < 0.01), but these levels were higher than in wild type mice (p < 0.05). With respect to blood cholesterol levels, these were higher in untreated diabetic mice than in the wild type mice (207 ± 35 vs. 156.5 ± 4.3 mg/ml, p < 0.05); whereas curcumin treatment decreased blood cholesterol levels in diabetic mice (161.4 ± 4 mg/ml) as compared to untreated diabetic mice (p < 0.05). There were no significant differences on blood levels triglycerides among wild-type, untreated diabetic and curcumin-treated diabetic mice (227.7 ± 40.3, 297 ± 59 and 344.8 ± 27 mg/ml, respectively).

### Curcumin couples oxygen consumption

**Figure 2:**
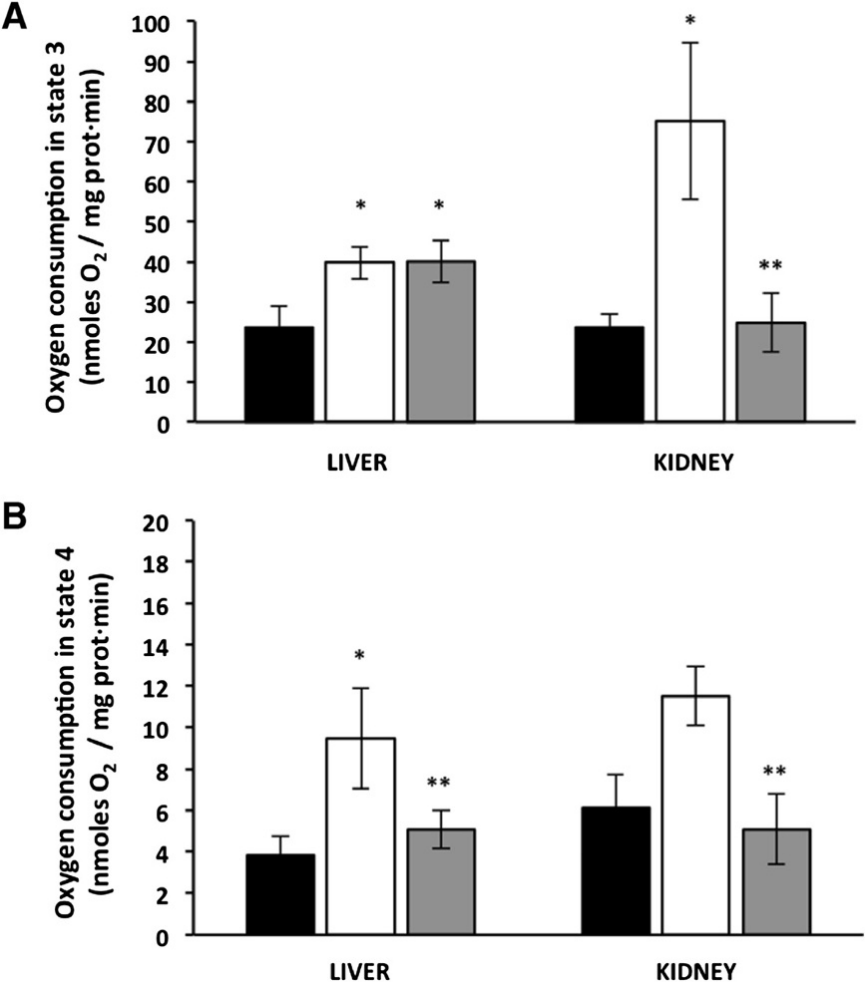
**Effect of hyperglycaemia and curcumin on mitochondrial respiration rate in state 3 (A) and state 4 (B).** Black bars, wild type mice (WTM, n = 4); empty bars, untreated-diabetic mice (UDM, n = 4); gray bars, treated-diabetic mice (TDM, n = 9). Data are given as the means ± standard error of the mean (SEM). *p < 0.05 vs. WTM; **p < 0.05 vs. UDM.

In liver mitochondria, state 3 respiration was similar between the diabetic untreated and curcumin-treated mice (40 ± 4.1 and 40 ± 5.2 nmoles O_2_/mg prot · min, respectively). There was a 66% increase in state 3 mitochondrial oxygen consumption in diabetic untreated and curcumin-treated mouse liver mitochondria compared to mitochondria derived from wt livers (24 ± 5.3 nmoles O_2_/mg prot · min) (Figure 2A). In mitochondria that were isolated from the diabetic untreated mouse livers, state 4 oxygen consumption was increased from 3.8 ± 0.9 to 9.4 ± 2.4 nmoles O_2_/mg prot · min, compared with mitochondria from the wt mouse livers, whereas state 4 respiration was 5 ± 0.9 nmoles O_2_/mg prot · min in mitochondria from the curcumin-treated mouse livers (Figure 2B); no differences were observed between diabetic curcumin-treated and wt mice. Furthermore, in curcumin-treated diabetic mice, state 4 oxygen consumption was decreased by 47% as compared with untreated diabetic mice.

The kidney was chosen because is one of the most frequently and severely affected organs. In addition, kidney is an insulin-independent organ in which glucose uptake is facilitated by the SGLT2 transporter, a protein that is abundant in the kidney compared to the liver [16]. As expected, in kidney mitochondria isolated from untreated diabetic mice, the state 3 oxygen consumption rate increased 3-fold compared with wt mice (75 ± 19.4 vs. 24 ± 3.5 nmoles O_2_/mg prot · min). In kidney mitochondria, curcumin treatment of the diabetic mice decreased state 3 oxygen consumption rates (25 ± 7.4 nmoles O_2_/mg prot · min) to levels that were similar to those obtained from the wild type mice (Figure 2A). In kidney mitochondria from untreated diabetic mice, state 4 oxygen consumption was two-fold higher compared with wt mice (11.5 ± 1.4 and 6.1 ± 1.6 nmoles O_2_/mg prot · min, respectively); whereas in kidney mitochondria isolated from diabetic mice, curcumin treatment restored state 4 oxygen consumption (5 ± 1.9 nmoles O_2_/mg prot · min) to levels similar to mitochondria isolated from wt mouse kidneys (Figure 2B).

Together, these results clearly demonstrate that the curcumin treatment decreased state 3 and 4 oxygen consumption in kidney mitochondria and only state 4 in liver.

### Curcumin increases ATPase activity in liver mitochondria

**Figure 3:**
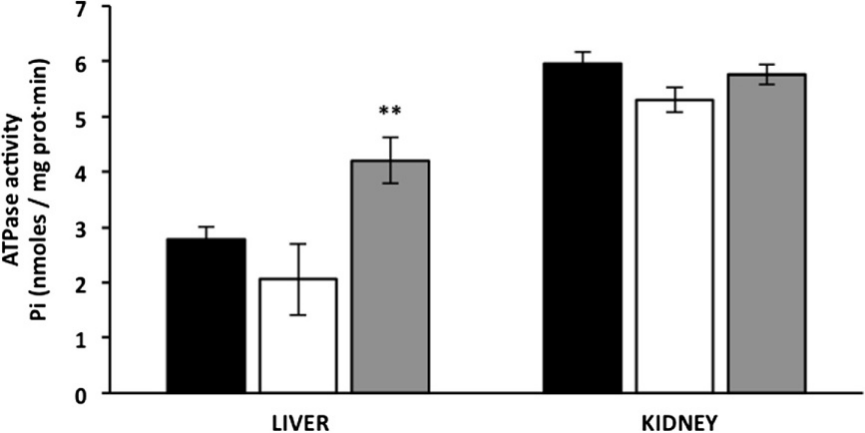
**Effect of hyperglycaemia and curcumin on mitochondrial ATPase activity.** Black bars, wild type mice (WTM, n = 4); empty bars, untreated-diabetic mice (UDM, n = 4); gray bars, treated-diabetic mice (TDM, n = 9). Data are given as the means ± standard error of the mean (SEM). **p < 0.05 vs. UDM.

Curcumin treatment decreased oxygen consumption in the liver and kidney; therefore we evaluated the effect of this polyphenol on ATPase activity. ATPase activity is an indirect measurement of the capacity that the mitochondria have to synthesis ATP. In liver mitochondria from untreated diabetic mice, ATPase activity was non-significantly reduced from 2.8 ± 0.24 to 2.05 ± 0.65 nmoles Pi/mg prot · min compared with liver mitochondria from wt mice, whereas curcumin treatment non-significantly increased liver mitochondria ATPase activity by 152% (4.2 ± 0.41 nmoles Pi/mg prot · min) compared with wt mice (Figure 3). Curcumin-treated diabetic mice had significantly higher liver mitochondria ATPase activity than untreated diabetic mice. Kidney mitochondrial ATPase activity was not significantly affected by hyperglycaemia or curcumin; thus, ATPase activity was similar between curcumin-treated diabetic, untreated diabetic and wild type mice (5.8 ± 0.17, 5.3 ± 0.23 and 6.0 ± 0.2 nmoles Pi/mg prot·min) (Figure 3).

### Curcumin restores nitric oxide synthesis in liver mitochondria

**Figure 4:**
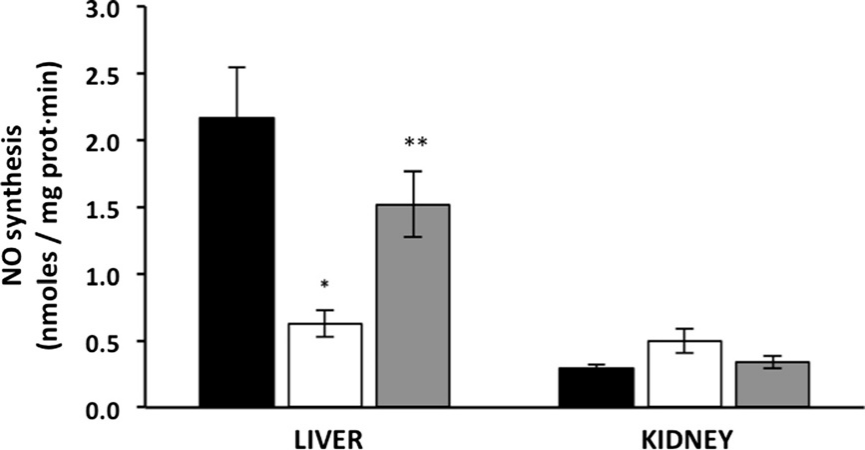
**Effect of hyperglycaemia and curcumin on mitochondrial nitric oxide synthesis.** Black bars, wild type mice (WTM, n = 4); empty bars, untreated-diabetic mice (UDM, n = 4); gray bars, treated-diabetic mice (TDM, n = 9). Data are given as the means ± standard error of the mean (SEM). *p < 0.05 vs. WTM; **p < 0.05 vs. UDM.

Mitochondrial NO regulates synthesis of ATP [17, 18] and is altered in diabetes [19]. Therefore, we determined the effect of curcumin on the synthesis of mitochondrial NO in *db/db* mice. NO synthesis was decreased in liver mitochondria from untreated diabetic mice compared with wt mice mitochondria (0.63 ± 0.1 vs. 2.17 ± 0.37 nmoles/mg prot · min, respectively) (Figure 4); however, in the treated diabetic mice, NO synthesis in liver mitochondria was only reduced by 21% (1.71 ± 0.31 nmoles/mg prot · min) compared with wt mice and was higher than in the untreated diabetic mice.

Kidney mitochondrial NO synthesis was non-significantly affected by diabetes or curcumin; thus, the values were 0.5 ± 0.1, 0.33 ± 0.05 and 0.28 ± 0.03 nmoles/mg prot · min in untreated diabetic, treated diabetic and wt mice, respectively (Figure 4).

### Curcumin decreases lipid peroxidation in liver and kidney mitochondria

**Figure 5:**
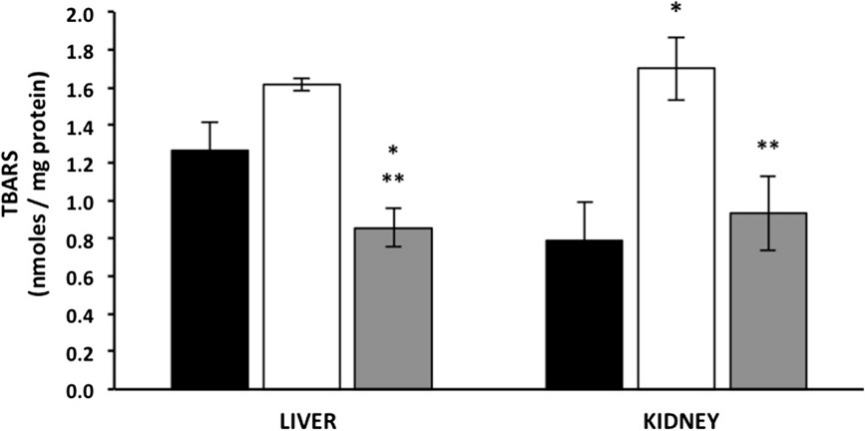
**Effect of hyperglycaemia and curcumin on TBARS levels.** Black bars, wild type mice (WTM, n = 4); empty bars, untreated-diabetic mice (UDM, n = 4); gray bars, treated-diabetic mice (TDM, n = 9). Data are given as the means ± standard error of the mean (SEM). *p < 0.05 vs. WTM; **p < 0.05 vs. UDM.

Figure 5 shows that in liver mitochondria isolated from the untreated diabetic mice, TBARS values were non-significantly higher than the wt mice (1.6 ± 0.35 and 1.27 ± 0.15 nmoles/mg protein, respectively), whereas curcumin treatment significantly decreased TBARS values (0.86 ± 0.1 nmoles/mg protein) in mitochondria from the diabetic mice compared with the wt and untreated-diabetic mice.

In kidney mitochondria, TBARS levels significantly increased in the untreated diabetic mice compared with the wt mice (1.7 ± 0.16 vs. 0.80 ± 0.2 nmoles/mg protein, respectively); however, in the curcumin-treated diabetic mice, TBARS levels were non-significantly higher than (0.93 ± 0.2 nmoles/mg protein) in the wt mice, and were significantly lower than in the untreated diabetic mice.

## Discussion

The *db/db* mouse model develop hyperphagic obesity and nonketotic diabetes similar to non-insulin-dependent diabetes mellitus in humans. Diabetic features in *db/db* mice follow an age-dependent progression, with early insulin resistance followed by an insulin secretory defect resulting in profound hyperglycemia. Thus, *db/db* mouse model has provided a useful resource to understand and treat the type 2 diabetic condition. For these reasons, in the present study, diabetic *db/db* mice were using as diabetes model.

The present results demonstrate that curcumin treatment significantly decreased body weight in *db/db* mice. It was reported that curcumin decreases body weight by reducing body fat and increasing lean mass in both high-fat-diet-induced obese and *ob/ob* mice [20].

Using mitochondria isolated from untreated *db/db* mice, we demonstrated that hyperglycaemia increases mitochondrial oxygen consumption in the liver and kidneys, compared with non-diabetic wild type mice (Figure 2). This finding contrasts with previous observation of mitochondrial dysfunction in liver from these same mice, which was characterized by a reduced respiratory capacity [21]. However, another study, *db/db* hearts exhibited reduced cardiac function and increased myocardial oxygen consumption; whereas mitochondrial ROS generation and lipid and protein peroxidation products were increased [22]. Thus, heart mitochondria from *db/db* mice exhibited fatty acid–induced mitochondrial uncoupling and increased expression of fatty acid oxidation genes and electron transfer flavoproteins, whereas the content of the F1 α-subunit of ATP synthase was reduced.

In kidney mitochondria from diabetic mice, curcumin treatment decreased state 3 and 4 oxygen consumption to levels that were similar to that in mitochondria from wild type mice (Figure 2). With respect to liver mitochondria, curcumin treatment only decreased the state 4 oxygen consumption to levels that were similar to that in mitochondria from wild type mice. It is important to consider that glucose uptake in the kidney mainly uses Glut1 transporter and that aerobic oxidation of glucose is the predominant pathway in the kidney; consequently, more redox equivalents are produced in the kidney [23].

We observed that ATPase activity was non-significantly lower in mitochondria from the liver and kidneys of the untreated diabetic mice, whereas treatment of these animals with curcumin significantly increased ATPase activity in the liver but not in the kidneys (Figure 3). Previous studies support that hyperglycaemia decreases ATP synthesis by causing changes in oxygen consumption both in humans and in rodents. For instance, obese and diabetic subjects have a low amount of mitochondrial and reduced capacity for mitochondrial ATP production in skeletal muscle [24–28]. Furthermore, hyperglycaemia decreased functions such as state 3 respiration, electron transport chain activity, ATP synthase activity, mitochondrial membrane potential, ROS production and oxidative damage in the heart of *db/db* mice [29, 30]. Thus, there is sufficient evidence that obesity and diabetes induce deficiencies in ATP synthesis [4–8, 24–28]. Therefore, the beneficial effect of curcumin treatment on mitochondrial functions suggests that this polyphenol could improve ATP production in the diabetic mouse liver (Figure 3), curcumin also decreases TBARS levels, suggesting that curcumin could reduce the free radicals production or remove these prooxidant molecules. Together, the present results suggest that curcumin increases the ratio of ATP production/oxygen consumption. However, in the present study, we did determine ATPase activity instead of ATP synthesis; for this reason, it is desirable to determine whether curcumin can increase ATP synthesis *in vivo*.

It has been reported that NO regulates oxygen consumption and consequently ATP production [17, 18]. Our results show that hyperglycaemia decreased NO synthesis in liver and weakly increased this synthesis in kidneys, whereas curcumin restored NO synthesis in both tissues (Figure 4). It is probable that curcumin restores NO synthesis and ATPase activity in liver by preventing the oxidation of the enzymes nitric oxide synthase (NOS) and ATPase or by inducing the expression of these enzymes. Thus, curcumin treatment of *db/db* mice restored NO synthesis and ATPase activity to wild type levels and also decreased oxygen consumption and lipid oxidation. Therefore, our data suggest that curcumin could be a good alternative to restore the altered mitochondrial NO production that is induced by hyperglycaemia in humans.

Because curcumin treatment restored oxygen consumption, ATPase activity and NO production, we evaluated whether treatment with this polyphenol reduced lipid peroxidation. We observed that diabetic mice had increased mitochondrial TBARS levels that were ameliorated by curcumin treatment both in the liver and in the kidneys (Figure 5). The liver has been demonstrated to have a higher oxidative metabolic rate than the kidneys and to also have increased levels of TBARS [31], which correlated with our results (Figure 5).

Taken together our results suggest that curcumin improves the rate of oxygen consumption as well as ATPase activity probably by scavenging free radicals and preventing protein and lipid oxidation. Given that central role for mitochondria is fuel utilisation and energy production, dysfunctional mitochondria, as are found in obesity and diabetes, can impact whole-body metabolic homeostasis. Mitochondrial uncoupling may be elevated in obesity due to increased FFA concentrations, which may result in the development of diabetes mellitus and/or other metabolic diseases [32, 33]. However, although the association between impaired mitochondrial function and diabetes mellitus is strong, a causal pathogenic relationship remains uncertain. For these reasons, the results presented in this study are important to suggest that hyperglycaemia induces mitochondrial dysfunction, whereas curcumin treatment reverts the lipid oxidation and mitochondrial dysfunction in the liver and kidney of *db/db* mice that is caused by hyperglycaemia. Our data are consistent with those reported previously in which administration of Resveratrol to *db/db* mice normalised mitochondrial function and biogenesis [34]. Moreover, curcumin protected brain mitochondria by direct detoxification as well as prevention of 3-nitrotyrosine formation in a mouse model of Parkinson’s disease [35, 36].

## Conclusions

Our findings suggest that in *db/db* mice, dietary supplementation with curcumin diminished mitochondrial dysfunction, likely by decreasing lipid peroxidation, which results in an increase of ATPase activity, restoration of oxygen consumption and NO synthesis in mitochondria isolated from the liver and kidneys of *db/db* mice. Together, these findings suggest that curcumin could be a good alternative to prevent or decrease nitrosative and oxidative stress as well as mitochondrial dysfunction during obesity and diabetes. Therefore, more research is necessary to elucidate whether curcumin may be effective for prevention and/or treatment of oxidative stress and mitochondrial dysfunction in obese and diabetes humans.

## Methods

### Animal use and care

Female and male heterozygote non-diabetic *db*/+ mice (BKS.Cg-*m* +/+ *Lepr*^*db*^/OlaHsd; Black, Lean) were purchased from Harlan Laboratories (Mexico, DF) and were back-crossed to start our colony. All mice had access to water and food (Harlan Laboratories; Cat. No. T.2018S.15) *ad libitum*. All animal procedures were performed in accordance with current Mexican legislation (NOM-062-ZOO-1999) and the National Institutes of Health (NIH, Bethesda, MD) Guide for the Care and Use of Laboratory Animals.

### Mouse genotypes and curcumin treatment

The mouse genotyping was performed as previously reported with some modifications [37–39]. Briefly, 1 mm of ear tissue was digested in 100 μl lysis buffer [100 mM Tris HCl (pH 8.5), 5 mM EDTA, 0.2% SDS, 200 mM NaCl, and 4 μg proteinase K/ml] overnight at 65°C with agitation, followed by incubation at 95°C for 10 min. Then 100 μl 7.5 M ammonium acetate (pH 2) was added to the lysate prior to centrifugation at 12,000 *g* for 15 min. The supernatant was recovered and mixed with 300 μl isopropanol followed by centrifugation at 12,000 *g* for 15 min. The precipitated DNA was washed with 500 μl ethanol, followed by centrifugation at 12,000 *g* for 15 min. Finally, the DNA was dissolved in 25 μl of water, and 1 μl of the DNA was used as the template for a 12.5 μl PCR reaction with the PCR master kit following the manufacturer’s instructions (ROCHE, Mannheim, Germany). Each primers was used at a concentration of 0.2 μM (forward, 5′-ATGACCACTACAGATGAACCCAGTCTAC-3′; reverse, 5′-CATTCAAACCATAGTTTAGGTTTGTCT-3′), as was previously reported [37–39]. The PCR conditions were as follows: an initial denaturation of 3 min at 95°C followed by 5 cycles of 95°C for 1 min, 60°C for 1 min, and 72°C for 30 sec; then 30 cycles of 92°C for 15 sec, 50°C for 1 min, and 72°C for 30 sec. Ten microliters of the PCR reaction was digested with the restriction enzyme AccI according to the manufacturer’s instructions (New England Biolabs, Inc.), followed by electrophoresis on a 15% polyacrylamide gel. Digestion with AccI yielded 85- and 24-bp fragments in the wild type (+/+) mice, 85-, 58-, 27-, and 24-bp fragments in heterozygote (*db/*+) mice, and 58-, 27-, and 24-bp fragments in the diabetic (*db/db*) mice.

Fourteen-week-old male mice were selected and grouped by genotype. Then 60 mg/kg curcumin was orally administered daily in the morning to 9 *db/db* mice for 4 weeks; 4 *db/db* and wt mice were used for sham controls, they received vehicle. Curcumin was dissolved in saline solution and administered using a stainless steel oral cannula. Curcumin was purchased from Sigma-Aldrich (70% purity; Cat. No. 1386. Saint Louis, MO, USA). At the end of the treatment, mice were sacrificed by cervical dislocation; then blood levels of glucose, triglycerides and cholesterol were determined using a commercial kit (Accutrend® GCT, Roche).

### Isolation of mitochondria

Liver and kidney mitochondria were prepared by differential centrifugation as we previously described [15].

### Oximetry assays

Oxygen consumption was measured at 25°C with an Oxytherm oxygen monitor (Hansatech Instruments Ltd; Norfolk, England) using 0.5 mg/ml mitochondrial protein in 1 ml of oximetry media as we previously described [15]. Briefly, for liver mitochondria, the oximetry media contained 120 mM KCl, 20 mM MOPS, 0.5 mM EGTA, 5 mM NaH_2_PO_4_ · H_2_O, and 10 mM NaCl, pH 7.4. For kidney mitochondria, the oximetry media contained 120 mM KCl, 2 mM EGTA, 10 mM HEPES, 5 mM KH_2_PO_4_ · H_2_O, and 1 mM MgSO_4_, pH 7.4. In the presence of 15 mM succinate and 2.2 mM rotenone, oxidative phosphorylation (oxygen consumption in state 3) was stimulated with 0.2 mM and 0.1 mM adenosine diphosphate (ADP) for liver and kidney mitochondria, respectively; then oxygen consumption in state 4 was measured when ADP was consumed.

### Measurement of NO production

NO production was determined spectrophotometrically by recording the reduction of oxyhaemoglobin (HbO_2_) by NO to methaemoglobin at 401 nm as we previously described [15].

### Determination of ATPase activity

ATPase activity was determined spectrophotometrically by recording the release of Pi from ATP [18]. Briefly, 8 μg of mitochondrial protein in 100 μl of reaction buffer (20 mM Tris-base, 5 mM MgCl2 and 0.1 mM ATP-Tris) were incubated at 25°C for 10 min, followed by addition of 10 μl of 30% trichloroacetic acid. Then 90 μl of water and 10 μl of 10% sodium dodecyl sulphate (SDS) were added, followed by addition of 25 μl of ammonium molybdate solution [11.1 ml of concentrated sulfuric acid and 5 g of molibdato ((NH_4_)_6_Mo_7_O_24_ · H_2_O) in 100 ml of water]. Next, 25 μl of ELON solution [0.1 g of 4-(methylamino)phenol hemisulfate salt and 0.3 g of sodium metabisulfite (Na_2_S_2_O_5_) in 10 ml of water] were added, followed by incubation at 25°C for 15 min. The absorbance was recorded at 660 nm and the amount of Pi was determined by a concentration standard curve, using sodium phosphate as standard.

### Measurement of lipid peroxidation

Reactive aldehyde levels were quantified with the thiobarbituric acid-reactive substances (TBARS) assay as we previously described [15].

### Statistical analysis

Data were analysed with ANOVA followed by Duncan’s multiple range tests. Data are represented as the means ± standard error of the mean (SEM). Values were considered statistically significant if *p* < 0.05.
